# Loss of LIN-35, the *Caenorhabditis elegans *ortholog of the tumor suppressor p105Rb, results in enhanced RNA interference

**DOI:** 10.1186/gb-2006-7-1-r4

**Published:** 2006-01-20

**Authors:** Ben Lehner, Andrea Calixto, Catriona Crombie, Julia Tischler, Angelo Fortunato, Martin Chalfie, Andrew G Fraser

**Affiliations:** 1 The Wellcome Trust Sanger Institute, Hinxton, Cambridge CB10 1SA, UK; 2 Department of Biological Sciences, Columbia University, New York, NY 10027, USA

## Abstract

**Background:**

Genome-wide RNA interference (RNAi) screening is a very powerful tool for analyzing gene function *in vivo *in *Caenorhabditis elegans*. The effectiveness of RNAi varies from gene to gene, however, and neuronally expressed genes are largely refractive to RNAi in wild-type worms.

**Results:**

We found that *C. elegans *strains carrying mutations in *lin-35*, the worm ortholog of the tumor suppressor gene *p105Rb*, or a subset of the genetically related synMuv B family of chromatin-modifying genes, show increased strength and penetrance for many germline, embryonic, and post-embryonic RNAi phenotypes, including neuronal RNAi phenotypes. Mutations in these same genes also enhance somatic transgene silencing via an RNAi-dependent mechanism. Two genes, *mes-4 *and *zfp-1*, are required both for the vulval lineage defects resulting from mutations in synMuv B genes and for RNAi, suggesting a common mechanism for the function of synMuv B genes in vulval development and in regulating RNAi. Enhanced RNAi in the germline of *lin-35 *worms suggests that misexpression of germline genes in somatic cells cannot alone account for the enhanced RNAi observed in this strain.

**Conclusion:**

A worm strain with a null mutation in *lin-35 *is more sensitive to RNAi than any other previously described single mutant strain, and so will prove very useful for future genome-wide RNAi screens, particularly for identifying genes with neuronal functions. As *lin-35 *is the worm ortholog of the mammalian tumor suppressor gene *p105Rb*, misregulation of RNAi may be important during human oncogenesis.

## Abstract 2

Mutations in lin-35, the worm ortholog of a mammalian tumor suppressor gene, and other synMuv B genes result in an increased sensitivity to RNAi and enhanced somatic transgene silencing.

## Background

Introduction of double-stranded RNA (dsRNA) into metazoan cells results in the sequence specific degradation of messenger RNA in a process known as RNA interference (RNAi) [1]. Components of the RNAi machinery are also involved in the regulation of endogenous gene expression, for example, in the silencing of repetitive DNA sequences and in the processing of microRNAs [2]. RNAi has proved a very powerful tool to examine gene function [3]. In *Caenorhabditis elegans*, RNAi is now routinely used to systematically examine *in vivo *loss of function phenotypes on a genome-wide scale. The efficiency of RNAi in *C. elegans *varies from gene to gene, however, such that the observed RNAi phenotype often does not represent the true null phenotype of a gene. In particular, genes expressed in neurons appear largely refractory to RNAi, which has precluded the use of RNAi screens to identify genes with neuronal functions [4]. For this reason there has been great interest in identifying worm strains that display an enhanced sensitivity to RNAi. Previously, mutations in two genes have been shown to enhance RNAi sensitivity in *C. elegans*. These genes are predicted to function in dsRNA synthesis or turnover, and encode a putative RNA-dependent RNA polymerase (*rrf-3 *[5]) and a ribonuclease (*eri-1 *[6]). A genome-wide RNAi screen in an *rrf-3 *mutant strain identified 393 additional genes with RNAi phenotypes because of its increased sensitivity to RNAi [7]. Here we report that inactivation of a subset of genes that function in the LIN-35/p105Rb chromatin-remodelling pathway also result in RNAi hypersensitivity in *C. elegans*. Indeed, we found that a straincarrying a null allele in *lin-35 *is more sensitive to RNAi than either *rrf-3 *or *eri-1 *mutant animals, making this strain an invaluable resource for future genome-wide RNAi screens.

## Results and discussion

### Loss of LIN-35 results in enhanced RNAi

The loss of function phenotypes generated by RNAi, like those generated by classic genetics, are highly dependent on the genetic background - many genes have very different RNAi phenotypes in a wild-type worms from those seen in animals mutant for a specific gene. Such genetic interactions can provide insight into how genes are organised into pathways. To begin to map out genetic interactions in the signal transduction and transcriptional networks that underpin *C. elegans *development, we used RNAi to individually target approximately 1,700 genes and compare the phenotypes generated in wild-type animals with the phenotypes in each of about 40 mutant strains; each strain carries a mutation in a key signalling component or chromatin regulator (B.L, C.C, J.T, A.F and A.G.F, manuscript submitted). The approximately 1,700 genes targeted encode the great majority of genes involved in signal transduction and transcriptional regulation, as annotated in Kamath *et al*. [4] (Additional data file 1). During this screening, we noticed that the RNAi phenotypes of many genes that had weak phenotypes in wild-type animals were greatly enhanced in the strain *lin-35(n745)*; this carries a putative null mutation in the *p105Rb *ortholog, *lin-35 *[8]. In this strain, the sterility and/or embryonic lethality of approximately 30% of all genes that had a weak phenotype in wild-type worms were enhanced (78 genes; Additional data file 2). Furthermore, 35 genes that had no detectable phenotype in wild-type worms had strong phenotypes in *lin-35 *mutants (Additional data file 2). In particular, many RNAi clones that result in partial F1 embryonic lethality in wild-type worms have complete P0 sterility or growth arrest in *lin-35(n745) *worms, suggesting a more rapid and complete inhibition of gene expression in the absence of *lin-35 *function.

The difference in RNAi phenotype for any gene that we observe in *lin-35(n745) *compared with wild-type could formally result either from an increase in RNAi sensitivity in the mutant or through some more complex genetic interactions (for example, through genetic buffering between *lin-35 *and a target gene). We believe the principal effect is through an increase in RNAi sensitivity for four reasons.

First, for genes that have a nonviable RNAi phenotype in *lin-35(n745)*, the genetic null allele is also always nonviable, when known (35 genes) (Additional data file 3), suggesting that the stronger phenotype represents a near-null state.

Second, for genes whose genetic nulls are viable but have distinct postembryonic phenotypes (for example, uncoordinated, dumpy), we also detect enhanced similar postembryonic phenotypes by RNAi (Table 1); this group of genes includes genes that affect the neuronal system, which is largely refractory to RNAi in wild-type animals [4].

**Figure 1:**
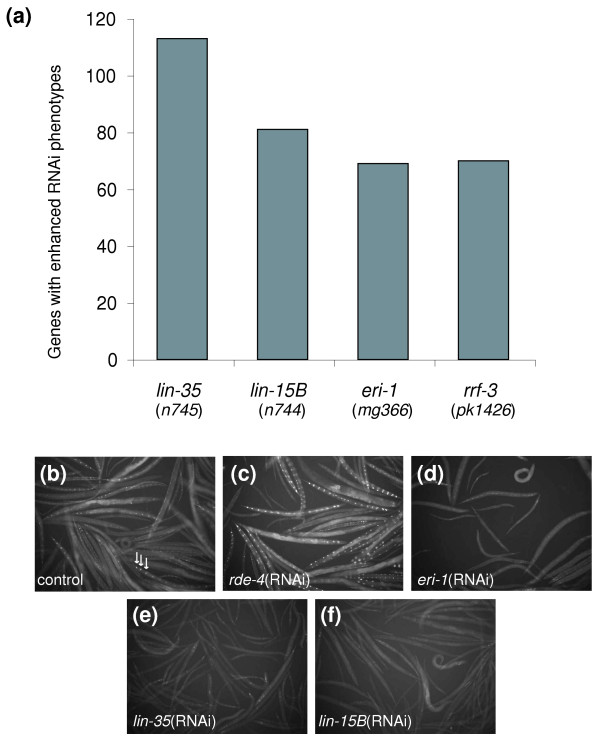
Inactivation of *lin-35 *or *lin-15B *enhances RNAi. **(a) **The number of genes with enhanced RNAi phenotypes in the worm strains *lin-35(n745)*, *lin-15B(n744)*, *eri-1(mg366) *and *rrf-3(pk1426)*. The chart shows the number of genes with RNAi phenotypes that are significantly stronger in each strain than in wild-type (Bristol N2) worms. A total of 1,838 bacterial RNAi feeding strains from the Ahringer library [4] targeting 1,749 genes were tested with each worm strain. **(b-f) **RNAi-induced silencing of *lin-35 *or *lin-15B *enhances the dsRNA-induced silencing of a GFP transgene. Worm strain GR1401 expresses an integrated GFP transgene and a dsRNA that targets GFP mRNA for degradation, both expressed specifically in the hypodermal seam cells of the worm (arrows) [9]. **(b) **Control experiments used a feeding strain that does not target any *C. elegans *gene and causes no change in the silencing of GFP. dsRNA targeting components of the RNAi machinery such as **(c) **rde-4 suppress the silencing of GFP, whereas dsRNAs targeting **(d) ***eri-1*, **(e) ***lin-35 *or **(f) ***lin-15B *result in enhanced silencing of GFP. See Table 2 for quantification of this data.

Thirdly, we tested whether inactivation of *lin-35 *increases gene silencing resulting from expression of an endogenously transcribed dsRNA. To do this, we took advantage of a system in which worms express GFP exclusively in the hypodermal seam cells, along with a dsRNA targeting the green fluorescent protein (GFP) mRNA [9]. In wild-type animals, where RNAi works with normal efficiency, there is a low level of GFP fluorescence in the seam cells due to targeting by the co-expressed dsRNA (54% of worms have GFP expression visible in their midbody seam cells; Figure 1b, Table 2). If RNAi is used to target genes required for RNAi, however, this reduces GFP knock-down, and there is an observed increase in GFP levels (for example, for *rde-4*, 67% of worms have GFP expression visible in their midbody seam cells; Figure 1c, Table 2); conversely, targeting genes whose loss increases RNAi efficiency results in a further reduction of GFP expression (for example, for *eri-1*, 13% of worms have GFP expression visible in their midbody seam cells; Figure 1d, Table 2; *p *< 0.001, Chi squared test). We found that targeting *lin-35 *causes a strong enhancement of GFP silencing in the seam cells (1% of worms have GFP expression visible in their midbody seam cells; Figure 1e, Table 2; *p *< 0.001). When combined with the enhanced RNAi phenotypes described above, this result is consistent with a model in which inactivation of *lin-35 *enhances the efficiency of RNAi. In addition, since in this system the dsRNA is expressed in the same cells in which the targeting occurs, we conclude that inactivation of *lin-35 *must enhance the cellular process of RNAi-induced gene silencing, rather than just altering the uptake or systemic transport of dsRNA. Taken together, these results indicate that mutations in *lin-35 *cause an increase in the effectiveness of RNAi and that this results in stronger and more penetrant RNAi phenotypes for many genes, making *lin-35(n745) *an invaluable research tool. We note that similar findings were reported by the Ruvkun lab while this manuscript was in preparation [10].

Finally, although inactivation of LIN-35 results in RNAi hypersensitivity, it is possible that some of the genes with an enhanced phenotype in *lin-35(n745) *animals could represent genetic interactions between *lin-35 *and a target gene via a mechanism that is independent of the RNAi hypersensitivity of this strain. To directly identify these genes, we took advantage of a strain carrying a mutation in a *lin-35 *pathway gene that does not show an increased sensitivity to RNAi. In both mammals and worms, LIN-35/Rb proteins are proposed to function by directly binding E2F family proteins [11,12]. The strain *efl-1(se1) *[13] carries a weakloss-of-function mutation in the worm E2F family gene *efl-1*, which is known to function with *lin-35 *in regulating cell-cycle progression [14], as well as development of the vulva [12] and pharynx [15]. *efl-1(se1) *animals do not show an increased sensitivity to RNAi, as judged by testing genes with an enhanced RNAi phenotype in *rrf-3(pk1426) *animals, or by inhibiting expression of *efl-1 *in the RNAi reporter strain GR1401. Thus, to identify genes that interact genetically with the *lin-35 *pathway, we tested whether genes that have an enhanced RNAi phenotype in *lin-35(n745) *animals, but not in *rrf-3(pk1426) *animals, also had enhanced RNAi phenotypes in *efl-1(se1) *animals (Additional data file 2). We found three genes that fulfilled these criteria (Table 3). The first of these genes is *pha-1*, which has previously been identified as genetically interacting with *lin-35 *and *efl-1 *[15], so validating the success of our approach. The other two genes represent novel *lin-35 *pathway genetic interaction partners: *dpy-22 *is predicted to encode a component of the mediator complex that, like LIN-35 and EFL-1, probably also functions in chromatin remodelling [16], and *Y106G6E.6 *encodes a Casein Kinase I family member. Intriguingly, targeting *Y106G6E.6 *by RNAi results in abnormalities in early embryonic polarity (C Panbianco and J Ahringer, personal communication); strong reduction of *efl-1 *function has previously been shown to affect embryonic polarity [13]. EFL-1 affects embryonic polarity at least in part through regulation of MAP kinase activity in the oocyte [13] and our data thus suggest that LIN-35, EFL-1, and Y106G6E.6 cooperate in some way to regulate MAPK activity in the *C. elegans *oocyte. There is no previously published functional association between p105Rb, E2F and a CKI family member and this underlies the strength of genetic interaction mapping as a way to reveal gene function.

### *lin-35 *animals are more sensitive to RNAi than previously described RNAi hypersensitive strains

We compared the RNAi sensitivity of animals carrying strong loss-of-function mutations in the two previously described genes that are known to negatively regulate RNAi in *C. elegans*, *rrf-3 *or *eri-1*, to that of *lin-35(n745) *animals. *rrf-3(pk1426)*[5] and *eri-1(mg366)*[6] enhanced the RNAi phenotypes of 70 and 69 of 1,749 genes tested, respectively, compared to 113 genes enhanced by *lin-35(n745) *(Figure 1a; Additional data file 2). Every gene displaying an increased phenotype with *rrf-3(pk1426) *or *eri-1(mg366) *also has an increased RNAi phenotype with *lin-35(n745)*. In addition, many genes that have enhanced RNAi phenotypes in *rrf-3(pk1426) *or *eri-1(mg366) *have even stronger phenotypes in *lin-35(n745)*.

Although the RNAi clones that we tested in each of the four strains represented a functionally biased set of genes, we also found very similar results when using random RNAi clones targeting genes with many diverse functions. In addition to the approximately 1,800 RNAi clones originally screened, we also screened the first 682 RNAi clones targeting genes on *C. elegans *chromosome III. These genes have very diverse molecular functions (Additional data file 4) and we found that 42 of these clones also had RNAi phenotypes that were stronger in *lin-35(n745) *than in *rrf-3(pk1426) *worms (Additional data file 5). In addition, it is not just the number of genes with enhanced RNAi phenotypes that is greater in *lin-35 *than in the other strains; the strengths of the RNAi phenotypes are also enhanced. For example, 11 of the genes we tested from chromosome III had an RNAi phenotype in *rrf-3 *worms that was further enhanced in *lin-35 *worms (Additional data file 5).

These results show that *lin-35(n745) *worms are more sensitive to RNAi than any previously described single mutant strain and are an ideal strain for new RNAi-based screens. This is a key finding - merely finding another hypersensitive strain is not a particularly useful research tool unless it is an improvement on the previously identified strains. Our ranking of the three strains is based on the use of a large set of test genes, and thus our conclusion is robust and not a curiosity of a few atypical RNAi phenotypes. We note, however, that Wang *et al*. [10] also provide evidence that a *lin-35(n745); eri-1(mg366) *double mutant strain may display a further enhancement in RNAi sensitivity to *lin-35(n745)*, suggesting that these two genes may partially function in parallel.

### *lin-35(n745) *animals display increased sensitivity to RNAi in the nervous system

For unknown reasons, many neuronally expressed genes appear largely refractory to RNAi in wild-type worms, precluding reverse genetic analyses [4]. We generated strong phenotypes for several neuronally expressed genes in *lin-35(n745) *animals (Table 1), suggesting RNAi-based screens for neuronal functions might be feasible in this strain. To test further for enhanced RNAi sensitivity in the nervous system of *lin-35(n745) *animals, we focused on genes expressed in the six touch receptor neurons of *C. elegans*. These neurons sense gentle touch to the body, and several mechanosensory abnormal (*mec*) genes have been identified that are needed for their development or function [17,18]. Although RNAi has been detected in these neurons when dsRNA is injected into animals [19], it is not seen when dsRNA is delivered by feeding in wild-type animals (AC, C Keller, and MC, unpublished data), rendering high-throughput RNAi screens impractical.

We tested the touch sensitivity of wild-type and *lin-35(n745) *animals fed on bacteria targeting eight *mec *genes (*mec-2*, *mec-3*, *mec-4*, *mec-8*, *mec-9*, *mec-10*, *mec-12 *and *mec-18*) and two unrelated genes (*gfp *and *sym-1*). In wild-type worms, none of the bacterial strains caused touch insensitivity - that is, the Mec phenotype - either in adults that had fed on the bacteria throughout their entire larval development or in their progeny (n > 30 for each). Thus, if bacterial-mediated RNAi is having an effect in the touch neurons of wild-type animals, the effect is too small to generate a detectable phenotype. In contrast, in parallel experiments, *lin-35 *adults that had been fed with bacteria targeting *mec-2*, *mec-3*, *mec-4*, *mec-9 *and *mec-18 *throughout their larval development were touch insensitive, although the animals displayed the Mec phenotype with differences in penetrance and expressivity. Penetrance ranged from 47% (*mec-9*) to 83% (*mec-2*). Bacteria expressing *mec*-*2*, *mec-3*, and *mec*-*4 *dsRNA consistently gave a highly penetrant phenotype with strong expressivity (that is, the animals had a touch insensitivity similar to animals with null alleles). Bacteria making dsRNA for *mec-12 *produced a highly penetrant phenotype (63%) with intermediate strength (the animals responded to a few touches). *mec-18 *bacteria produced less consistent but easily detectable results; in some experiments the penetrance was high (60%) and expressivity strong, whereas in others the penetrance was lower (45%) and the expressivity intermediate. Bacteria producing *mec*-9 dsRNA gave the weakest positive results with penetrance of 47% and intermediate expressivity. These weaker effects seen with *mec-9*, *mec-12 *and *mec-18 *may be a consequence of the high expression of these genes in the touch neurons [20], which might overwhelm the RNAi machinery. Animals fed on bacteria targeting *mec-8 *or *mec-10 *were indistinguishable from those fed on bacteria for the *gfp *and *sym-1 *controls. Although negative RNAi results are difficult to interpret, genetic experiments [18] indicate that the amount of *mec-8 *activity produced in the embryo is sufficient for subsequent adult touch sensitivity, and elimination of *mec-10 *has only a slight effect on touch sensitivity (R O'Hagan, M Goodman, and MC, unpublished data).

These data indicate that neuronally expressed genes are effectively targeted by bacterial-mediated RNAi in the *lin-35(n745) *strain, thus providing a very useful tool to study gene function in these cells. These results also point to the expression and function of *lin-35 *in post-mitotic neurons.

### A subset of synMuv B genes negatively regulate RNAi and somatic transgene silencing

In addition to demonstrating the usefulness of the *lin-35(n745) *strain for generating enhanced RNAi phenotypes, we wished to explore the connection between *lin-35 *and RNAi. *lin-35 *encodes the *C. elegans *ortholog of the human tumour suppressor gene *p105Rb *and is, therefore, presumed to act as a chromatin regulator. Thus, while *rrf-*3 and *eri-1 *encode proteins that are intimately connected with dsRNA synthesis and turnover, no clear mechanistic link is known between *lin-35 *and RNAi, making the connection between chromatin remodelling and RNAi an intriguing question. *lin-35 *functions in the synthetic Multivulva (synMuv) B pathway that is redundantly required with the synMuv A pathway to antagonise the outcome of Ras signalling in the specification of vulval cell lineages [8]. Although some synMuv genes are of unknown molecular function, several synMuv B genes encode the worm orthologs of components of p105Rb transcriptional repressor complexes identified in mammals and flies [11,21-23]. If the chromatin remodelling function of LIN-35 is important for its effect on RNAi, one would anticipate that strains carrying mutations in othersynMuv B genes would also be hypersensitive to RNAi. To test the RNAi sensitivity of synMuv strains, we used the subset of bacterial feeding clones that gave an enhanced RNAi phenotype in *rrf-3(pk1426) *animals. We tested these clones for enhanced RNAi phenotypes in each of the synMuv strains compared to in wild-type worms. We found that strains carrying inactivating mutations in the synMuv B genes *lin-15B *(Figure 1a, Table 4), *dpl-1*, and *lin-9 *(Table 4) also enhanced the RNAi phenotypes of the majority of these genes. In addition, Sieburth *et al*. [24] have shown that an *eri-1*; *lin-15B *double mutant is also hypersensitive to neuronal RNAi phenotypes. In contrast, strong loss-of-function mutations in two other synMuv B genes, *lin-36 *and *tam-1*, or the synMuv A gene *lin-15A *did not enhance any RNAi phenotypes for these genes (Table 4). We conclude that a subset of synMuv B genes negatively regulate RNAi, which we refer to as synMuv B(R) genes. Wang *et al*.[10] obtained similar results.

**Figure 2:**
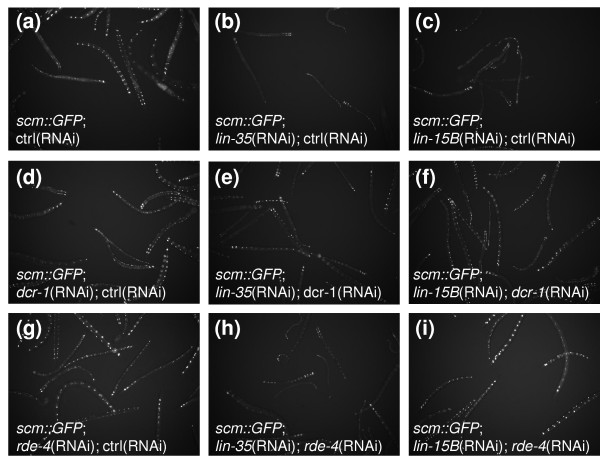
*lin-35 *and *lin-15B *enhance somatic transgene silencing by an RNAi dependent mechanism. Worm strain JR667 expresses GFP specifically in the hypodermal seam cells from an integrated tandemly repeated array of the construct wIs51, which contains the *scm::GFP *reporter **(a)**. Inactivation of either *lin-35 ***(b) **or *lin-15B ***(c) **results in enhanced silencing of the GFP transgene via a mechanism that is dependent upon the RNAi machinery, including *dcr-1 ***(e-f) **and *rde-4 ***(h-i)**. Inactivation of *dcr-1 ***(d) **or *rde-4 ***(g) **alone results in a slight increase in GFP expression, indicating a background level of transgene silencing in wild-type worms. Worms were fed on 1:1 mixes of the indicated RNAi feeding strains and RNAi feeding experiments were performed as described in Materials and methods. Control (ctrl) RNAi experiments used the same non-targeting RNAi clone as used in Figure 1. See Table 5 for quantification of this data.

In addition to increasing sensitivity to RNAi, inactivation of the genes *rrf-3 *or *eri-1 *also results in the silencing of somatically expressed transgene tandem arrays via an RNAi-dependent mechanism [9]. Consistent with their having roles as negative regulators of the RNAi pathway, inactivation of synMuv B(R) genes also results in somatic transgene silencing [25] (data summarised in Tables 4 and 5 and Figure 2b,c). In contrast, inactivation of other synMuv B or synMuv A genes does not result in somatic transgene silencing [25] (Table 4). Somatic transgene silencing in animals with inactivated synMuv B(R) genes can be suppressed by inactivation of components of the RNAi machinery (Figure 2e,f,h,i, Table 5). Thus we conclude that inactivation of synMuv B(R) genes induces somatic transgene silencing as a result of an increase in RNAi. We also note, however, that mutations in at least one other synMuv B gene, *tam-1*, can enhance somatic transgene silencing [25], but without any observable effect on RNAi sensitivity (Table 4). This suggests that other genes may be able to enhance transgene silencing independently of the RNAi pathway via an unknown mechanism. Indeed, we found that RNAi of *dcr-1 *does not suppress transgene silencing in a strain carrying a mutation in *tam-1 *(reduced transgene silencing was seen in 0 of >300 *tam-1(cc567) *[25] animals tested (Additional data file 6)). The general picture is clear, however: the subset of synMuv B genes that affects RNAi sensitivity is very similar to the subset that alters transgene silencing, suggesting that these form a genetically distinct group of synMuv B genes.

Additional evidence suggests this subclassification of synMuv B genes is functionally relevant. Inactivation of a subset of synMuv B genes results in ectopic expression of a *lag-2::gfp *reporter gene [26]. Strikingly, all of the synMuv B genes that we found to be negative regulators of the RNAi pathway and negative regulators of somatic transgene silencing also negatively regulate *lag-2::gfp *expression [26] (Table 4). This result suggests a similar synMuv B(R) pathway may regulate both the RNAi pathway and correct expression of this transgene, and supports the classification of synMuv B genes into at least two distinct functional subsets.

### Genes required for RNAi can suppress the lineage defects of synMuvA;B strains

**Figure 3:**
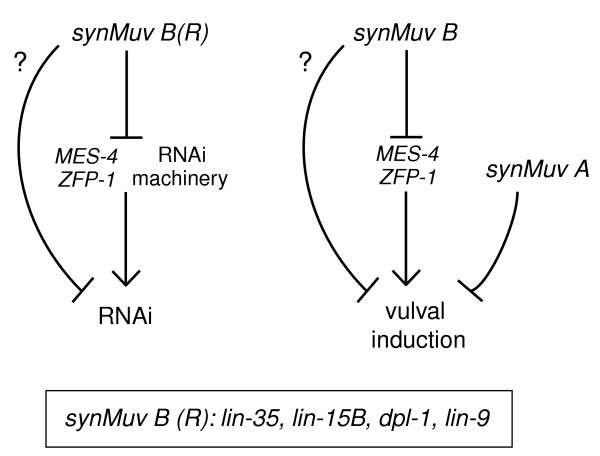
A comparison of the genetic pathways that regulate RNAi and vulval development. A subset of the synthetic Multivulva B genes, designated the synMuv B(R) genes, negatively regulate vulval induction (redundantly with the synMuv A pathway), and also negatively regulate somatic and germline RNAi. In both processes the genes *mes-4 *and *zfp-1 *act genetically downstream of, or in parallel to, the synMuv B(R) genes. The identities of the synMuv B(R) genes are given below the figure.

Combined mutations in both a synMuv A and a synMuv B gene (for example, in the *lin-15A;B (n765) *strain) results in the development of ectopic vulvae (the so-called multivulva or Muv phenotype). In an RNAi screen for genes that can suppress the Muv phenotype of a *lin-15A;B(n765) *strain, we identified two genes, *mes-4 *and *zfp-1*, that have been previously identified as necessary for both RNAi and somatic transgene silencing (Table 6; C.C and A.G.F, unpublished data) [9,27]. These genes both have clear human orthologs, and are both predicted to encode components of chromatin modifying complexes: *mes-4 *encodes a putative trithoraxgroup histone methyltransferase protein with a SET domain and three PHD finger domains and is orthologous to human *WHSC1L1*, and *zfp-1 *encodes a PHD finger domain protein and is orthologous to human *MLLT10/AF10*, a fusion partner of *MLL *(a *mes-4 *related gene) in acute leukaemia [28]. The requirement of *mes-4 *and *zfp-1 *for RNAi and their ability to suppress mutations in synMuvA;B mutants, suggests that a common mechanism may underlie the role of synMuv B genes in these two processes (Figure 3).

### An increase in RNAi efficiency does not cause the lineage defects of synMuv B mutants

The precise molecular functions of the synMuv B(R) genes and of *mes-4 *and *zfp-1 *in vulva development and in RNAi are unknown; however many of these genes are predicted by sequence homology to regulate chromatin structure. One intriguing possibility is that a key function of the synMuv B(R) genes during vulva development may be to repress RNAi. The Muv phenotype might thus be due in part to alterations in RNAi-related processes. We investigated this in two complementary ways. Firstly, if the sole effect of synMuv B(R) genes on vulval development was through their effect on RNAi sensitivity, then other genes that similarly increase RNAi sensitivity should act as synMuv B genes. However, while targeting *lin-35 *by RNAi produces a strong Muv phenotype in a *lin-15A *mutant animal (as expected given its synMuv B activity), targeting *eri-1 *has no similar effect. Secondly, inactivation of the synMuv B(R) genes enhances RNAi and in the absence of synMuv A activity leads to multivulval development; to determine if these two functions were causally related, we asked whether inactivation of other genes that are essential for RNAi (*rde-1*, *rde-4*, *rde-5*, *mut-7 *or *mut-16*) suppresses the Muv phenotype of *lin-15A;B(n765) *- they do not. Hence, we find that genes that enhance RNAi do not all act as synMuv B genes and, conversely, that the RNAi machinery is not necessary for the synMuv phenotype. Thus, alterations in the efficacy of RNAi cannot alone account for the action of *lin-35 *in vulval development, although it may contribute to *lin-35*'s role.

Wang *et al*. [10] suggest that the enhanced RNAi seen in synMuv B mutants may result from the misexpression of germline genes in somatic cells. Although this may contribute to the enhanced somatic RNAi seen in synMuv B strains, we found that *lin-35(n745) *animals also showed enhanced germline RNAi phenotypes (>50 genes gave strong sterility in *lin-35(n745) *but not in wild-type worms) (Additional data file 2). Although some of these sterile phenotypes may result from defects in somatic cells, a subset of these genes has been previously shown to function within the germline itself. In *C. elegans*, the Notch and MAP kinase pathways are both required within the germline for correct germline development [29,30], and we found that four genes that function in these pathways also show strongly enhanced RNAi-induced sterility in *lin-35(n745) *worms (the genes *glp-1*, *lag-1*, *let-60 *and *lin-45*; Additional data file 3). Since the enhanced sterility seen with these genes must result from enhanced gene silencing within the germline itself, these data demonstrate that RNAi is also enhanced in the germline of *lin-35(n745) *worms, and that somatic misexpression of germline genes does not alone account for the enhanced RNAi seen in synMuv B mutants. We favour a model in which the synMuv B(R) genes and *mes-4/zfp-1 *act antagonistically to regulate the expression of a common set of target genes. These targets could include genes that are required for vulval development and genes required for RNAi, or the genes targeted by RNAi themselves. The antagonism may involve the direct repression of *mes-4 *and *zfp-1 *by the synMuv B(R) genes, or the antagonistic action of *mes-4/zfp-1 *and the synMuv B(R) genes on a common set of target genes (Figure 3). Alternatively, MES-4/ZFP-1 and the synMuv B(R) gene products may antagonise each other's functions by competing for a common set of co-factors.

## Conclusion

We have found that *lin-35 *and a subset of synMuv B pathway genes negatively regulate RNAi in *C. elegans*, probably via a mechanism involving chromatin remodelling. The efficiency of RNAi is enhanced within both somatic and germline cells of *lin-35 *animals, demonstrating that misexpression of germline genes in somatic cells cannot alone account for the enhanced RNAi seen in this strain. *lin-35(n745) *is the most RNAi-sensitive single mutant strain identified to date and, therefore, should prove very useful for genome-wide RNAi screens. We note that the availability of five strains with varying RNAi-sensitivities (*lin-35(n745) *> *lin-15B(n744) *> *eri-1(mg366) *approximately = *rrf-3(pk1426) *> *N2*; Figure 1a) opens the possibility of studying an 'allelic series' of RNAi phenotypes for many genes (for example, when L1 wild-type or *lin-15B(n744) *worms are fed on bacteria targeting the gene *ftt-2*, they reach adulthood, at which point wild-type worms have a reduced brood size while the *lin-15B(n744) *worms are completely sterile; the RNAi phenotype is so severe in *lin-35(n745) *worms, however, that the L1 worms never reach adulthood, and instead show a completely penetrant larval growth arrest). We have also identified two genes (*mes-4 *and *zfp-1*) that are both required for RNAi and can suppress the vulval lineage defects resulting from inactivation of synMuv genes, suggesting a common mechanism for the action of synMuv B(R) genes in both of these processes. However, the increased efficiency of RNAi in synMuv B mutants does not alone explain the lineage defects of synMuv B strains.

Finally, it is possible that the human ortholog of LIN-35, p105Rb, may also negatively regulate RNAi, and its effect on the RNAi pathway may be important for its function as a tumour suppressor. In addition, inactivation of the human orthologs of *mes-4 *or *zfp-1 *may reverse some of the phenotypic consequences of mutations in *p105Rb*.

## Materials and methods

### RNAi screens by bacterial feeding

All of the RNAi feeding experiments described in this manuscript were performed in liquid culture by adding synchronised L1 stage worms, unless otherwise indicated. A total of 1,868 bacterial RNAi feeding strains from the Ahringer library [4] targeting 1,749 genes were tested with each worm strain. The vast majority of these genes are those annotated as 'signalling', 'chromatin', or 'transcription factors' in reference [4] (Additional data file 1); feeding approximately 75% of these bacterial strains gave no visible RNAi phenotypes in wild-type worms [4]. Bacterial RNAi feeding strains were grown overnight at 37°C in 400 μl 2TY plus 100 μg/ml ampicillin, induced with 4 mM IPTG (isopropyl-beta-D-thiogalactopyranoside) at 37°C for 1 hour, and resuspended in 400 μl NGM(Nematode Growth Medium) plus 4 mM IPTG plus 100 μg/mlampicillin. Approximately 10 L1 stage worms were dispensed to each well of a 96-well flat bottomed tissue culture plate together with 40 μl of resuspended bacterial culture. The plates were incubated with shaking at 20°C for four days, and embryonic lethal, sterile, growth defect and post-embryonic phenotypes were scored on a dissecting microscope. All RNAi feeding experiments were performed in quadruplicate and the phenotypes observed in each strain were directly compared to those seen in N2 worms grown in parallel.

### Transgene silencing assays

Worm strain JR667 expresses GFP specifically in the hypodermal seam cells from an integrated tandemly repeated array of the construct *wIs51*, which contains the *scm::GFP *reporter. Worm strain GR1401 expresses the same integrated GFP transgene, as well as a dsRNA that targets GFP mRNA for degradation, also specifically in the hypodermal seam cells of the worm [9]. RNAi experiments were performed on six-well plates seeded with the indicated bacterial RNAi feeding strain (grown overnight as described above). Approximately 10 L1 stage worms were added to each well and incubated at 25°C for four days, unless otherwise indicated. The progeny worms were washed off the plates, paralysed in 100 mM levimasole and GFP expression was visualised using an Olympus IX81 microscope. Control experiments used a feeding strain that does not target any *C. elegans *gene (constructed using primers sjjY95B8A_84.g, defined in [31]). All other RNAi assays were performed exactly as described in [4].

## Additional data files

The following additional data are available with the online version of this paper. Additional data file 1 provides the complete set of genes screened by RNAi feeding. Additional data file 2 lists genes with an enhanced RNAi phenotype in each of four RNAi hypersensitive strains, and in *efl-1(se1)*. Additional data file 3 lists genes with nonviable RNAi phenotypes in *lin-35(n745) *that also have nonviable null phenotypes. Additional data file 4 lists genes from chromosome III tested for enhanced RNAi phenotypes in the strains *lin-35(n745) *and *rrf-3(pk1426)*. Additional data file 5 lists genes from the start of chromosome III with an enhanced RNAi phenotype in *lin-35(n745) *compared to *rrf-3(pk1426)*. Additional data file 6 is a figure showing that loss of *dcr-1 *does not suppress somatic transgene silencing resulting from inactivation of *tam-1*.

## Supplementary Material

Additional data file 1 The complete set of genes screened by RNAi feeding. Click here for file

Additional data file 2 Genes that have an enhanced RNAi phenotype in each of the hypersensitive strains or in the non-hypersensitive strain *efl-1(se1) *are indicated. Genes with a weak RNAi phenotype in wild-type (N2) worms are also indicated, together with the RNAi phenotype in *lin-35(n745) *animals. Emb, embryonic lethal; Gro, growth arrest of parental generation resulting in an F1 brood size of 0; Red, reduced brood size; Ste, sterile. Click here for file

Additional data file 3 Genes with nonviable RNAi phenotypes in *lin-35(n745) *that also have nonviable null phenotypes. Click here for file

Additional data file 4 Genes from chromosome III tested for enhanced RNAi phenotypes in the strains *lin-35(n745) *and *rrf-3(pk1426)*. Gene 'functional class' descriptions are taken from [4]. Click here for file

Additional data file 5 682 RNAi clones targeting genes from the start of chromosome III were fed to *lin-35(n745)*, *rrf-3(pk1426) *and wild-type worms. Forty-two clones had an RNAi phenotype that was stronger in *lin-35(n745) *than in *rrf-3(pk1426)*. Of these, 11 had an RNAi phenotype in *rrf-3 *that was further enhanced in *lin-35*. 0, no visible phenotype; Adl, adult lethal; Emb, embryonic lethal (scored on a semi-quantitative scale from 0 to 3); Gro, growth arrest in first generation; Lvl, larval lethal in first generation; Red, reduced brood size; Ste, sterile. Click here for file

Additional data file 6 Strain PD6249 expresses a *myo-3::GFP *transgene in all muscle cells. Expression of the transgene is partially silenced at 20°C due to a mutation in the gene *tam-1 *[25]. RNAi against **(a) **a control gene, or **(b) ***dcr-1 *has no effect on the level of transgene silencing (in contrast to silencing resulting from loss of *lin-35*, which is lost if components of the RNAi pathway are inactivated; Figure 2). Click here for file

## Figures and Tables

**Table 1:** Enhanced post-embryonic RNAi phenotypes observed in *lin-35(n745) *worms

RNAi	N2 phenotype*	*lin-35(n745) *phenotype
*dpy-13*	Dpy +	Dpy +++
*unc-104*	Unc +	Unc +++
*unc-18*	WT	Unc

*RNAi feeding experiments were performed at 20°C, as described in [4]. Dpy, dumpy; Unc, uncoordinated; WT, wild-type. +, weak phenotype; ++, medium phenotype; +++, strong phenotype.

**Table 2:** RNAi induced silencing of *lin-35 *or *lin-15B *enhances the dsRNA-induced silencing of a GFP transgene

RNAi*	Complete^†^	GFP expression partial^‡^	None^§^	% Complete expression
*control*	44	15	23	54%
*lin-35*	1	25	75	1%
*lin-15B*	1	16	78	1%
*eri-1*	7	16	30	13%
*rde-4*	28	6	8	67%

*RNAi experiments were performed as described in Materials and methods. ^†^Number of worms with GFP expression in all hypodermal seam cells. ^‡^Number of worms with GFP expression only in their anterior and/or posterior seam cells, but not in the midbody seam cells. ^§^Number of worms with no GFP expression visible in any cells.

**Table 3:** Identification of genes that genetically interact with the *lin-35 *pathway

RNAi	Enhanced RNAi phenotype		
	
	*efl-1(se1)*	*lin-35(n745)*	*rrf-3(pk1426)*
*pha-1*	Yes	Yes	No
*dpy-22*	Yes	Yes	No
*Y106G6E.6*	Yes	Yes	No

**Table 4:** A subset of synMuv B genes negatively regulate RNAi, somatic transgene silencing and expression of *lag-2::gfp*

Gene	synMuv	Enhanced RNAi*	Somatic transgene silencing^†^	Ectopic expression of *lag-2::gfp*^‡^
*lin-35(n745)*	B	Yes	Yes	Yes
*lin-15B(n744)*	B	Yes	Yes	Yes
*lin-9(n112)*	B	Yes	Yes	Yes
*dpl-1(n3643)*	B	Yes	nd	Nd
*lin-36(n766)*	B	No	No	No
*tam-1(cc567)*	B	No	Yes	nd
*lin-15A(n767)*	A	No	No	No
*lin-15A;B(n765)*	AB	Yes	nd	nd

*RNAi sensitivity was determined as described above, but using the subset of bacterial feeding clones that gave an enhanced RNAi phenotype in *rrf-3(pk1426)*. ^†^Somatic transgene silencing data are taken from [25]. ^‡^*lag-2::gfp *expression data are taken from [26]. nd, not determined.

**Table 5:** *lin-35 *and *lin-15B *enhance somatic transgene silencing by an RNAi dependent mechanism

RNAi*	GFP expression		% Complete expression
		
	Complete^†^	Partial^‡^	
*control*	27	2	93%
*dcr-1; control*	15	0	100%
*rde-4; control*	19	0	100%
*lin-35; control*	1	11	8%
*lin-35; dcr-1*	11	18	38%
*lin-35; rde-4*	14	17	45%
*lin-15B; control*	2	14	13%
*lin-15B; dcr-1*	30	2	94%
*lin-15B; rde-4*	25	5	83%

*RNAi experiments were performed as described in Materials and methods. ^†^Number of worms with GFP expression in all hypodermal seam cells. ^‡^Number of worms with GFP expression only in their anterior and/or posterior seam cells, but not in the midbody seam cells.

**Table 6:** *mes-4 *and *zfp-1 *suppress the multivulval phenotype of *lin-15A;B(n765) *worms

Genotype	Multivulval*
*lin-15A;B(n765);control(RNAi)*	100% (n = 300)
*lin-15A;B(n765); mes-4(RNAi)*	5% (n = 172)
*lin-15A;B(n765); zfp-1(RNAi)*	46% (n = 96)

*RNAi experiments were performed at 20°C, as described in [4].
