# Supplementary material for: Loss of LIN-35, the Caenorhabditis elegans ortholog of the tumor suppressor p105Rb, results in enhanced RNA interference
Source: Genome Biol. 2006 Jan 20;7(1):R4. doi: 10.1186/gb-2006-7-1-r4 (PMC1431716; doi:10.1186/gb-2006-7-1-r4)
Supplement: Additional data file 3 — Genes with nonviable RNAi phenotypes in lin-35(n745) that also have nonviable null phenotypes. [file gb-2006-7-1-r4-s3.doc]

**Additional file 3.** Genes with nonviable RNAi phenotypes in *lin-35(n745)* that also have nonviable null phenotypes

| Gene^1^ | RNAi phenotype^1^ | |
| --- | --- | --- |
|  | *lin-35(n745)* | N2 |
| *ceh-32* | Emb | weak Emb |
| *chk-1* | Ste | weak Emb |
| *dli-1* | Ste | weak Emb |
| *ftt-2* | Ste | weak Ste |
| *glp-1* | Ste | weak Ste |
| *hbl-1* | Ste | medium Ste |
| *hlh-1* | medium Ste | Wt |
| *hsf-1* | Ste | Wt |
| *kin-18* | Emb/medium Ste | Wt |
| *lag-1* | Ste | Emb |
| *ldb-1* | Ste | Wt |
| *let-502* | medium Ste | weak Emb |
| *let-60* | medium Ste | Wt |
| *let-653* | medium Ste | Wt |
| *lin-3* | Ste | medium Ste |
| *lir-1* | Ste | weak Emb |
| *mat-3* | Emb | weak Emb |
| *mom-2* | Emb | weak Emb |
| *mom-5* | Emb | weak Emb |
| *mpk-1* | Ste | weak Ste |
| *mup-4* | Ste | medium Ste |
| *mys-1* | Ste | Wt |
| *nhr-25* | Ste | weak Ste |
| *nhr-67* | Ste | weak Ste |
| *odd-2* | medium Ste | Wt |
| *par-1* | Ste | weak Emb/weak Ste |
| *peb-1* | weak Emb/weak Ste | weak Emb |
| *pha-1* | Ste | Wt |
| *pqn-9* | Ste | weak Emb/weak Ste |
| *qua-1* | Emb/medium Ste | Wt |
| *sdc-2* | Emb | weak Emb |
| *sur-6* | Emb/weak Ste | weak Emb |
| *trr-1* | Ste | weak Emb |
| *unc-120* | Ste | weak Ste |
| *unc-52* | Ste | Wt |

^1^The table lists all of the genes with a nonviable RNAi phenotype in *lin-35(n745)* for which a genetic null mutation is available. The genetic nulls of all of these genes are nonviable.

^2^ WT – wild-type, Emb – 100% embryonic lethal, weak Emb – <100% embryonic lethal, Ste – brood size 0, medium Ste – brood size very reduced, weak Ste – brood size slightly reduced.
