# Supplementary figures and images for: Loss of LIN-35, the Caenorhabditis elegans ortholog of the tumor suppressor p105Rb, results in enhanced RNA interference
Source: Genome Biol. 2006 Jan 20;7(1):R4. doi: 10.1186/gb-2006-7-1-r4 (PMC1431716; doi:10.1186/gb-2006-7-1-r4)

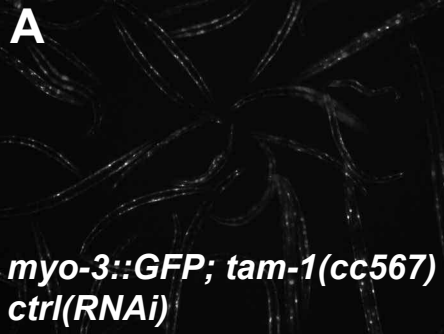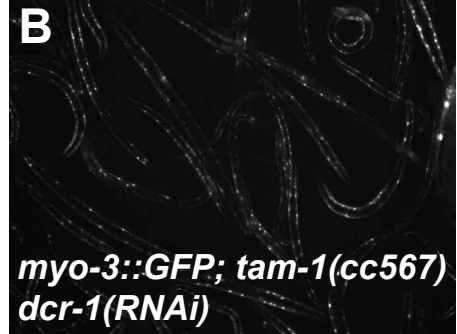

Supplement: Additional data file 6 — Strain PD6249 expresses a myo-3::GFP transgene in all muscle cells. Expression of the transgene is partially silenced at 20°C due to a mutation in the gene tam-1 [25]. RNAi against (a) a control gene, or (b) dcr-1 has no effect on the level of transgene silencing (in contrast to silencing resulting from loss of lin-35, which is lost if components of the RNAi pathway are inactivated; Figure 2). [file gb-2006-7-1-r4-s6.pdf]
